# Laboratory Puzzle of Oxidative Stress, Parameters of Hemostasis and Inflammation in Hospitalized Patients with COVID-19

**DOI:** 10.3390/biomedicines12030636

**Published:** 2024-03-13

**Authors:** Jelena Djordjevic, Vesna Ignjatovic, Vladimir Vukomanovic, Katarina Vuleta, Nevenka Ilic, Zivana Slovic, Marijana Stanojevic Pirkovic, Olgica Mihaljevic

**Affiliations:** 1 Department of Pathophysiology, Faculty of Medical Sciences, University of Kragujevac, 34000 Kragujevac, Serbia; jeladj997@gmail.com; 2 Department of Nuclear Medicine, Faculty of Medical Sciences, University of Kragujevac, 34000 Kragujevac, Serbia; vesnaivladaignjatovic@gmail.com (V.I.); vukomanovic@gmail.com (V.V.); kvuleta87@gmail.com (K.V.); 3 University Clinical Center Kragujevac, 34000 Kragujevac, Serbia; zivanaminic@yahoo.com (Z.S.); marijanas14@gmail.com (M.S.P.); 4 Institute for Public Health Kragujevac, 34000 Kragujevac, Serbia; ilic.nena.nevenka@gmail.com; 5 Department of Forensic Medicine, Faculty of Medical Sciences, University of Kragujevac, 34000 Kragujevac, Serbia; 6 Department of Biochemistry, Faculty of Medical Sciences, University of Kragujevac, 34000 Kragujevac, Serbia

**Keywords:** COVID-19, oxidative stress, inflammation, hemostasis abnormalities

## Abstract

Bearing in mind that coronavirus disease (COVID-19) is associated with a wide range of laboratory abnormalities, the aim of this study was to examine the importance of determining the parameters of oxidative stress and antioxidant protection as well as markers of inflammation and hemostasis in hospitalized patients with COVID-19. The study population included 105 patients with severe COVID-19 and 65 healthy control subjects. The parameters of oxidative stress and the activity of enzymes of the antioxidant system were determined from the obtained samples using spectrophotometric methods. Standard laboratory methods were performed for the determination of the biochemical and hematological parameters. Patients with COVID-19 showed a significantly higher level of pro-oxidative parameters (hydrogen peroxide (H_2_O_2_) and the index of lipid peroxidation in the form of thiobarbituric acid-reactive substances (TBARSs)) and a significantly lower activity of the antioxidant system (catalase (CAT)). Patients with COVID-19 had significantly higher values of inflammation parameters (C-reactive protein (CRP), procalcitonin (PCT), ratio of the number of neutrophils to lymphocytes (NLR), and ratio of the number of platelets to lymphocytes (PLR)) and parameters of hemostasis (activated partial thromboplastin time (aPTT), prothrombin time (PT), D-dimer, fibrinogen) than the control healthy subjects. In addition, changes in hemostatic parameters correlated positively with inflammatory markers in the group of patients with COVID-19. The early determination of hemostasis parameters and the parameters of inflammation can help in the prediction of poor prognosis in COVID-19 patients.

## 1. Introduction

In a short period of time, severe acute respiratory syndrome coronavirus 2 (SARS-CoV-2) caused a worldwide pandemic [1]. Although the clinical features of coronavirus disease 2019 (COVID-19) are well defined, the laboratory abnormalities observed in suffering patients are still poorly understood [2,3,4].

Most COVID-19 infections are asymptomatic or mild. However, in a significant number of patients, the infection causes severe respiratory disease that requires hospitalization [5]. Uncontrolled SARS-CoV-2 infection can cause the excessive release of proinflammatory mediators, leading to multiorgan damage through hypercoagulability and oxidative stress [6]. Furthermore, under the cytokine storm caused by COVID-19, an abnormal level of oxidants is generated, leading to the oxidation of a large number of macromolecules and additional damage [7]. Additionally, numerous studies have indicated that oxidative stress triggers endothelial damage, further contributing to the cytokine storm and coagulopathy [8,9]. On the other hand, endothelial cells stimulated by proinflammatory cytokines may contribute to local oxidative stress, which in turn leads to endothelial dysfunction and an increased risk of complications in COVID-19 patients [10].

Although thrombosis and inflammation have long been considered to be separate physiological processes, an intense interdependence between these mechanisms has been recognized [11]. Platelets play an important role in the development of thrombotic processes but also represent an important bridge that mediates between the hemostatic system and the inflammatory response [12]. Moreover, platelets can interact with viruses and represent a source of numerous inflammatory mediators, thus contributing to the cytokine storm reported in COVID-19 [13]. Accordingly, inflammatory and infectious diseases are often associated with a prothrombotic response known as immunothrombosis [12,13]. The thrombo-inflammatory process triggered by an excessive systemic inflammatory response could significantly affect vascular endothelium damage, abnormal clot formation, and excessive activation of the coagulation system and platelets [14,15]. Many patients with COVID-19 have been shown to have a high prevalence of laboratory abnormalities that increase the risk of coagulopathy [16]. Also, severe or fatal cases of COVID-19 were associated with higher levels of inflammatory markers compared to milder cases [17,18]

The main goal of this research was to analyze the parameters of oxidative stress and antioxidant protection in hospitalized patients with COVID-19, as well as their association with markers of inflammation and parameters of hemostasis. We also evaluated the differences in the investigated parameters between patients with COVID-19 and healthy controls.

## 2. Materials and Methods

### 2.1. Study Population

The study included 105 patients of both genders suffering from COVID-19, hospitalized at the University Clinical Center Kragujevac in the period from January 2021 to the beginning of May 2021. There were 65 (61.9%) men and 40 (38.1%) women, with a mean age of 59.6 ± 14.78 years, in whom SARS-CoV-2 was confirmed by real-time polymerase chain reaction (PCR). The study included only subjects who had not been vaccinated before the infection with COVID-19. All patients met the criteria of the World Health Organization for COVID-19 [19]. The study did not include children under the age of 18, pregnant women, patients with corneal and autoimmune diseases, immunocompromised patients, patients with malignant diseases (on chemotherapy), patients with coagulation disorders, as well as those who used antioxidant supplementation before admission.

**Figure 1:**
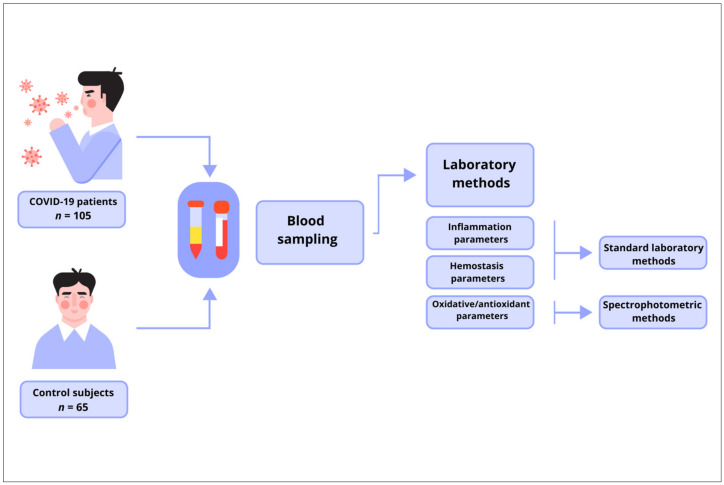
Schematic diagram summarizing the methodologies used in the study.

The control group consisted of 65 healthy subjects, 42 (64.6%) men and 23 (35.4%) women with an average age of 58.91 ± 11.71 years. The group comprised colleagues who were ready to join our research team and who did not suffer from COVID-19, nor were they vaccinated. All control subjects tested negative for coronavirus antigen and did not develop symptoms of COVID-19. All parameters examined in the study at the time of sampling were within the reference range. In addition, the healthy subjects did not have acute or chronic infections, autoimmune diseases, malignant diseases, coagulation disorders, or other conditions that could affect the investigated parameters (Figure 1).

The study was conducted according to the Declaration of Health, and it was approved by the Ethics Committee of the University Clinical Center Kragujevac (number 01/21-138). Informed consent for participation in the study was obtained from all patients and control subjects.

### 2.2. Determination of Redox Status

#### 2.2.1. Blood Sampling

Whole-blood samples were taken after venipuncture. Blood was collected in commercially available Vacutainer tubes containing 3.2% sodium citrate. Then, the blood was centrifuged for 10 min at 3000 rpm to separate plasma and erythrocytes. The plasma was stored in a freezer at −80 °C. The isolated erythrocytes were washed three times with a cold physiological solution, and then, 3 mL of distilled water was added to 1 mL of erythrocytes (centrifugal force was 1912× *g*).

Oxidative stress parameters were determined spectrophotometrically in plasma by measuring superoxide anion radical (O_2_^−^), hydrogen peroxide (H_2_O_2_), nitric oxide (NO) in the form of nitrites (NO_2_^−^) and the index of lipid peroxidation in the form of thiobarbituric acid-reactive substances (TBARSs).

The status of antioxidant protection was determined spectrophotometrically in erythrocyte lysates by measuring the selected antioxidant enzymes superoxide dismutase (SOD), catalase (CAT) and reduced glutathione (GSH).

#### 2.2.2. Measurements of Oxidative Stress Parameters

##### Superoxide Anion Radical (O_2_^−^) Determination

The nitro blue tetrazolium (NBT) reaction was used to determine the concentration of O_2_^−^. We pipetted 50 μL of the plasma sample and 950 μL of the prepared washing mixture into the test tubes. The absorbance was measured three times while stirring with a plastic rod. The level of O_2_^−^ was measured at the wavelength of maximum absorption λmax = 550 nm. The corresponding volume of distilled water was used as a blank [20].

##### Hydrogen Peroxide (H_2_O_2_) Determination

The determination of the H_2_O_2_ level was based on the oxidation of phenol red by H_2_O_2_ in a reaction catalyzed by the horseradish peroxidase (HRPO) enzyme. The procedure involved pipetting 800 μL of freshly prepared phenol red solution (PRS) and 10 μL of HRPO prepared ex tempore into 200 μL of plasma sample. The concentration of released H_2_O_2_ in the plasma sample was calculated based on the calibration diagram (standard curve). After 10 min of incubation at room temperature, the absorbance at λ = 610 nm was measured. An equivalent volume of distilled water was used as a blank [20].

##### Nitrite (NO_2_^−^) Determination

Considering that nitrogen monoxide (NO) decomposes quickly, the level of NO was estimated by indirect measurement of the level of NO_2_^−^. The spectrophotometric method for the biochemical determination of nitrate is based on the use of Griess regens. Amounts of 1 μL of plasma, 250 μL of freshly prepared Griess reagent (forms a purple diazo complex) and 125 μL of ammonia buffer were pipetted into the test tubes. The prepared mixture was placed on ice for 15 min and then centrifuged at 6000 rpm. After pouring off the supernatant, 220 μL of potassium carbonate (K_2_CO_3_) was added. The measurement was performed at λ = 550 nm. An equivalent volume of distilled water was used as a blank [20].

##### Determination of the Index of Lipid Peroxidation

The degree of lipid peroxidation in plasma was assessed indirectly by measuring the level of lipid peroxidation reaction products with thiobarbituric acid (TBARS). First, 200 μL of 1% TBA dissolved in 0.05 sodium hydroxide with 800 μL of plasma sample was incubated in a water bath for 15 min at 100 °C. After incubation, the samples were left for 10 min at room temperature, and the measurement was performed at λ = 530 nm. An appropriate volume of distilled water was used as a blank [20].

#### 2.2.3. Measurements of Antioxidant Parameters

##### Determination of Superoxide Dismutase (SOD) Activity

The Beutler method of epinephrine was used to evaluate SOD activity. An amount of 100 μL of erythrocyte lysate with 1000 μL of carbonate buffer was pipetted into the tubes, and after a few seconds in the Vortex mixer, 100 μL of epinephrine was added. Absorption was measured spectrophotometrically at λ = 470 nm. Distilled water was used as blank instead of blood lysate [20].

##### Determination of Catalase (CAT) Activity

The procedure for determining CAT activity included Aebi spectrophotometric monitoring of the rate of N_2_O_2_ decomposition in the presence of catalase. Amounts of 50 μL of CAT buffer, 100 μL of the prepared lysate and 1000 μL of 10 mM H_2_O_2_, which initiates the reaction, were pipetted into the test tubes. The measurement was performed six consecutive times at λ = 360 nm. An equivalent volume of distilled water was used as blank instead of blood lysate [20].

##### Determination of Reduced Glutathione (GSH) Level

The level of GSH was determined according to the Beutter method, i.e., by oxidation of GSH with 5,5-dithiobis-6,2-nitrobenzoic acid (DTNB). A total of 50 μL of lysate was pipetted with 200 μL of 0.1% ethylenediaminetetraacetic acid (EDTA) and 385 mL of percipitated buffer, then placed on ice for 15 min and centrifuged for 15 min. An amount of 300 μL of the obtained extract was added to a test tube with 750 μL of dibasic sodium phosphate and 100 μL of DTNB. After 10 min of incubation, the measurement was performed using the spectrophotometric method at λ = 420 nm. An equivalent volume of distilled water was used as a blank [20].

### 2.3. Determination of Hematological and Biochemical Parameters

In the Laboratory Diagnostic Service of the University Clinical Center Kragujevac, biochemical parameters were determined using standard accepted methods. C-reactive protein (CRP) and procalcitonin concentrations were measured by reagents on an Oly AU 680 (Beckman Coulter Inc., Brea, CA, USA) for CRP, and a Cobas e 411 chemical analyzer (Roche diagnostics GmbH, Mannheim, Germany) for procalcitonin. Reference ranges were as follows: CRP < 5 mg/L and procalcitonin < 0.5 ng/mL.

Hematological parameters of the hemoglobin level (range 138–175 g/L for males and 110–157 g/L for females), hematocrit (0.415–0.530 L/L for males and 0.356–0.470 L/L for females), blood count of erythrocytes (range 4.34–5.72 × 10^12^/L for males and 3.86–5.08 × 1012/L for females), leucocytes (3.70–10.0 × 10^9^/L), absolute number of leukocyte subtypes (range 2.10–6.50 × 10^9^/L for neutrophil granulocytes), lymphocytes (range 1.20–3.40 × 10^9^/L), platelets (135–450 × 10^9^/L), mean platelet volume (MPV) (range 6.8–10.4 fl) and platelet distribution width (PDW) (ratio 12.0–16.5) were measured using the automated DxH 800 Hematology Analyzer (Beckman Coulter, Inc. Brea, CA, USA). Based on the mentioned measurement, the ratio of the absolute number of neutrophil leukocytes and lymphocytes (NLR) and the ratio of the absolute number of platelets and lymphocytes (PLR) were calculated.

ACL TOP 350CTS (Beckman Coulter Inc. Brea, USA) was used to evaluate hemostasis parameters (prothrombin time (PT), activated partial thromboplastin time (aPTT), D-dimer and fibrinogen). The reference range of coagulation parameters was as follows: PT 11.8–15.3 s; aPTT 25–35 s; D-dimer < 0.50 μg/mL; and fibrinogen 2–5 g/L.

### 2.4. Statistical Analysis

All data were statistically analyzed using SPSS version 20.0 for Windows. The results are expressed as mean ± standard deviation. To assess the difference in the analyzed parameters between the two groups of subjects, the *t*-test of an independent sample (parametric) or the Mann–Whitney test (non-parametric) was used. The relationship between the variables was checked by bivariate correlation test with the determination of the Pearson/Spearman coefficient.

The heatmap was plotted by https://www.bioinformatics.com.cn/en (accessed on 10 March 2024). A *p* value less than 0.05 was considered statistically significant.

## 3. Results

The study included 105 hospitalized patients with COVID-19, 65 (61.9%) men and 40 (38.1%) women, with an average age of 59.6 ± 14.78 years. The second study group consisted of 65 healthy subjects, 42 (64.6%) men and 23 (35.4%) women, with an average age 58.91 ± 11.71 (Table 1).

**Figure 2:**
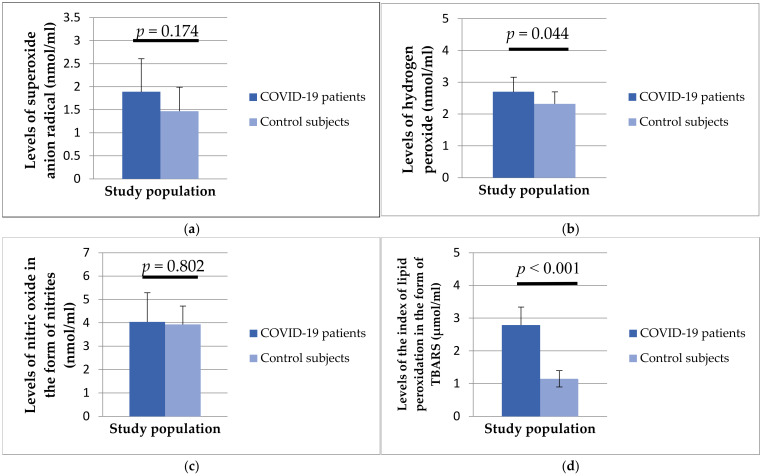
Values of superoxide anion radical (**a**), hydrogen peroxide (**b**), nitric oxide in the form of nitrite (**c**), and lipid peroxidation index in the form of TBARS (**d**) in patients with COVID-19 and healthy subjects.

Figure 2 shows the level of oxidative stress parameters in the plasma samples of the studied population. A higher level of oxidative stress was found in patients with COVID-19 compared to healthy subjects, and H_2_O_2_ (2.70 ± 0.46 vs. 2.32 ± 0.38 nmol/mL, *p* = 0.44) and TBARS (2.79 ± 0.55 vs. 1.15 ± 0.25 μmol/mL, *p* < 0.001) showed a statistically significant difference between the examined groups.

**Figure 3:**
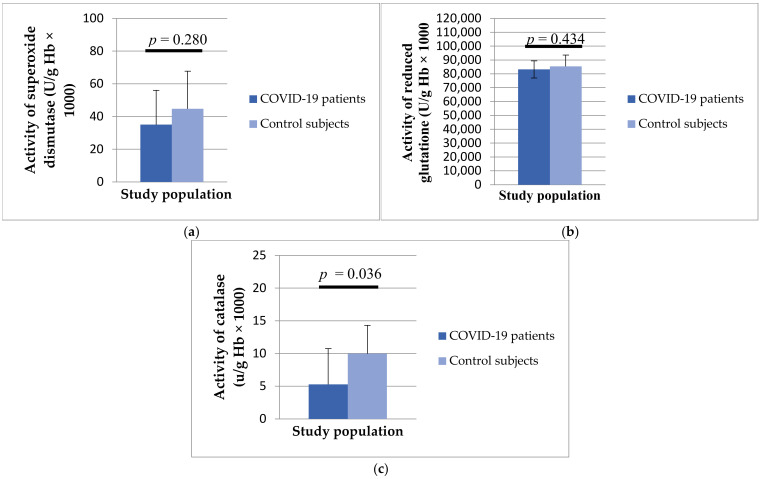
Values of superoxide dismutase (**a**), catalase (**b**) and reduced glutathione (**c**) in patients with COVID-19 and healthy subjects.

Regarding antioxidant enzymes, the activity of SOD (35.03 ± 21.03 vs. 44.77 ± 23.02 U/g Hb × 10^3^, *p* = 0.280), GSH (83,199.58 ± 8245.14 vs. 85,394 ± 6188.39 U/g Hb × 10^3^, *p* = 0.434) and CAT (5.28 ± 4.34 vs. 2.35 ± 5.47 U/g Hb × 10^3^, *p* = 0.036) was statistically lower in patients with COVID-19 compared to healthy controls (Figure 3).

Our study included the analysis of certain hematological and biochemical parameters in patients with COVID-19 and healthy controls (Table 2).

**Figure 4:**
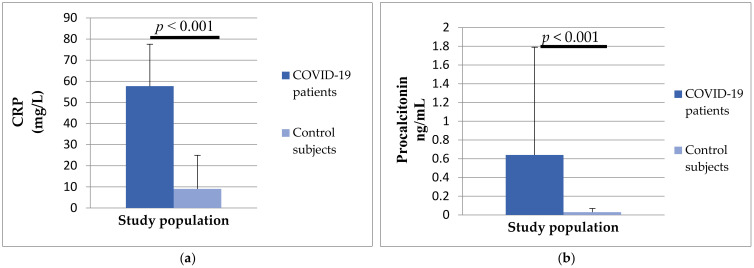
The differences in CRP (**a**) and procalcitonin (**b**) concentrations between patients with COVID-19 and control subjects.

Analyzing the serum concentrations of inflammation parameters, it was found that there was a difference between the two investigated groups. Patients with COVID-19 had a significantly higher concentration of CRP (57.66 ± 19.9 vs. 9.05 ± 15.9, *p* < 0.001) and procalcitonin (0.64 ± 1.57 vs. 0.03 ± 0.04, *p* < 0.0019) compared to the control group of subjects (Figure 4).

**Figure 5:**
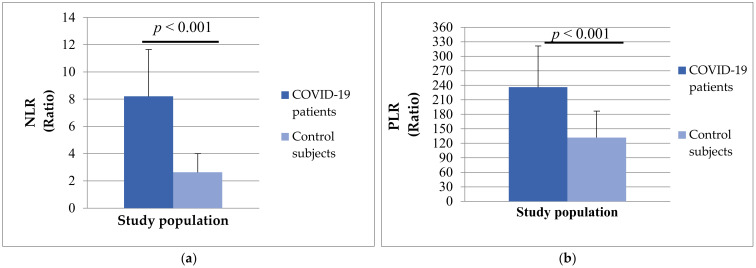
The differences in NLR ratio (**a**) and PLR ratio (**b**) between patients with COVID-19 and control subjects.

In addition, the group of patients with COVID-19 had significantly higher values of NLR (8.21 ± 13.45 vs. 2.63 ± 1.38, *p* < 0.001) and PLR (236.18 ± 185.13 vs. 131.89 ± 54.75, *p* < 0.001) compared to healthy subjects (Figure 5).

**Figure 6:**
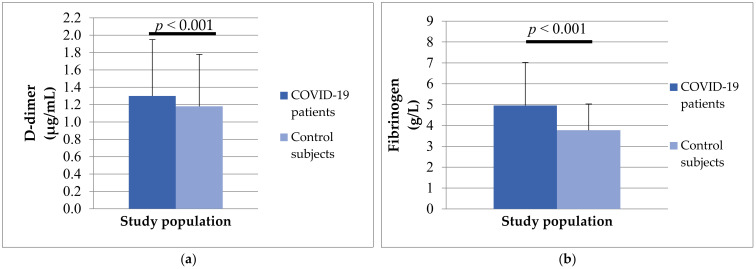
The differences in D-dimer (**a**) and fibrinogen (**b**) concentrations between patients with COVID-19 and control subjects.

Analyzing hemostasis parameters, we noticed that patients with COVID-19 had longer PT (13.47 ± 4.42 vs. 12.43 ± 1.96, *p* = 0.188) and aPTT (30.07 ± 4.62 vs. 28.26 ± 5.92, *p* = 0.070), but without significant differences. A statistically significant difference was obtained in the concentration of D-dimer (1.30 ± 1.65 vs. 1.18 ± 3.65) and fibrinogen (4.96 ± 2.06 vs. 3.77 ± 1.26) between the two study groups (Figure 6). The mean values of both analyzed parameters were significantly higher in patients with COVID-19 (*p* < 0.001).

**Figure 7:**
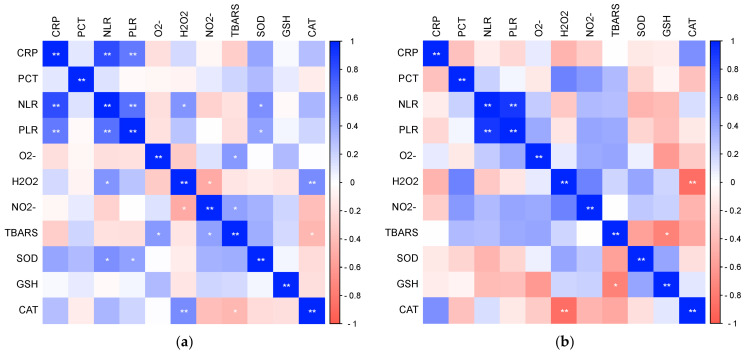
The ratio of pro-oxidative/antioxidant parameters with markers of inflammation in patients with COVID-19 (**a**) and healthy subjects (**b**) (* *p* ≤ 0.05, ** *p* ≤ 0.01).

**Figure 8:**
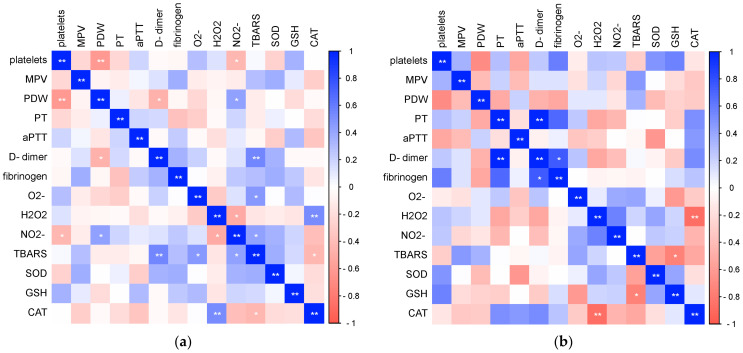
The ratio of pro-oxidative/antioxidant parameters with markers of hemostasis in patients with COVID-19 (**a**) and healthy subjects (**b**) (* *p* ≤ 0.05, ** *p* ≤ 0.01).

In further research, statistically significant relationships were found between oxidative stress and antioxidant protection with parameters of inflammation (Figure 7), as well as between oxidative stress and antioxidant protection with parameters of hemostasis (Figure 8) in patients with COVID-19. There was a significant positive relationship between H_2_O_2_ and NLR (Pearson, r = 0.481, *p* = 0.027) and a significant positive relationship between TRABS and D-dimer concentration (Spearman, r = 0.517, *p* = 0.016), while NO_2_^−^ statistically correlated significantly negatively with platelet count (Pearson, r = −0.430, *p* = 0.041) and statistically positively with PDW (Pearson, r = 0.421, *p* = 0.046). SOD activity showed a significant positive relationship with systemic inflammation markers NLR (Pearson, r = 0.523, *p* = 0.015) and PLR (Pearson, r = 0.434, *p* = 0.049). There were no significant associations in the healthy subjects.

**Figure 9:**
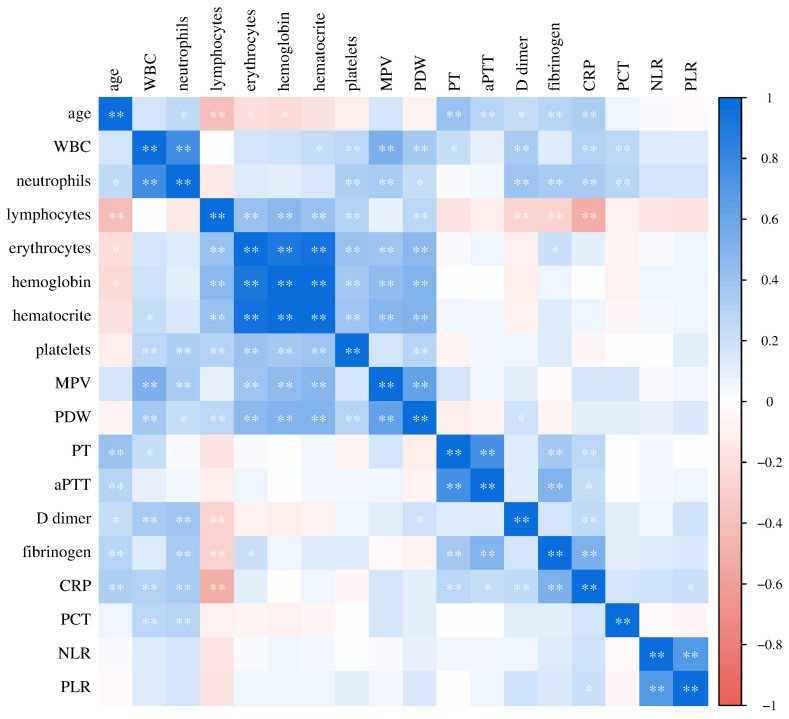
Relationship of laboratory parameters in COVID-19 patients (* *p* ≤ 0.05, ** *p* ≤ 0.01).

**Figure 10:**
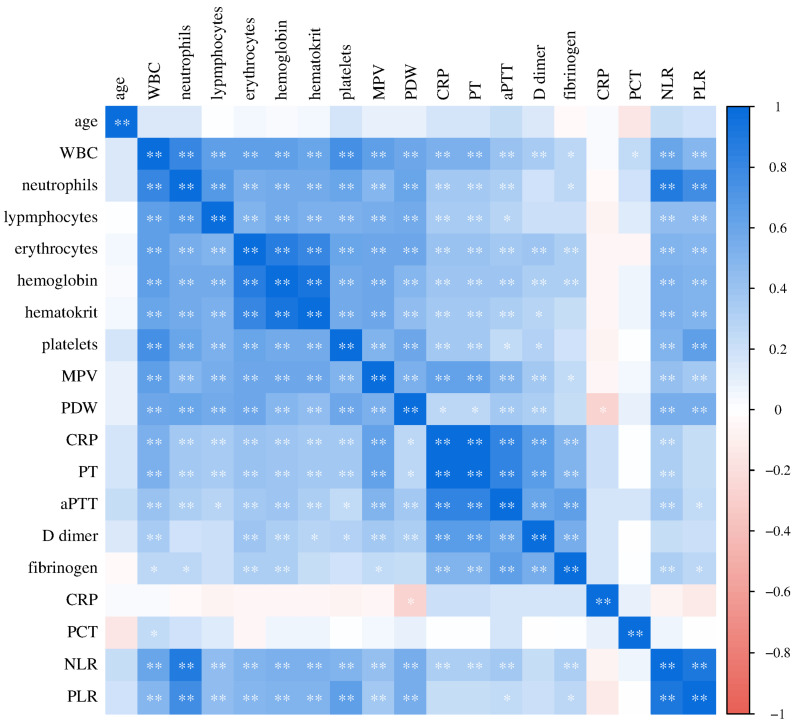
Relationship of laboratory parameters in the control group of subjects (* *p* ≤ 0.05, ** *p* ≤ 0.01).

Finally, we analyzed the relationships of the investigated laboratory parameters in patients with COVID-19 (Figure 9) and healthy controls (Figure 10). Bivariate correlation analysis confirmed the existence of a statistically significant positive relationship between CRP concentration and PT (Spearman r = 0.556, *p* < 0.001), aPTT (Pearson r = 0.379, *p* < 0.001), D-dimer concentration (Spearman r = 0.593, *p* < 0.001) and fibrinogen (Spearman r = 0.762, *p* < 0.001), as well as a statistically significant positive relationship of procalcitonin concentration with PDW (Spearman r = 0.329, *p* = 0.012), PT (Spearman r = 0.315, *p* = 0.017), aPTT (Pearson r = 0.269, *p* = 0.043), D-dimer concentration (Spearman r = 0.389, *p* = 0.003) and fibrinogen (Spearman r = 0.493, *p* < 0.001) in patients with COVID-19. Markers of systemic inflammation, NLR and PLR, also showed significant correlations with hemostasis parameters. NLR showed a significant positive relationship with MPV (Spearman r = 0.0.244, *p* = 0.016), PT (Spearman r = 0.551, *p* < 0.001), D-dimer concentration (Spearman r = 0.620, *p* < 0.001) and fibrinogen (Spearman r = 0.528, *p* < 0.001), while PLR positively correlated with platelet count (Pearson r = 0.268, *p* = 0.008), PT (Spearman r = 0.343, *p* < 0.001), D-dimer concentration (Spearman r = 0.465, *p* < 0.001) and fibrinogen (Spearman r = 0.494, *p* < 0.001).

In the control group of healthy subjects, a significant positive correlation of CRP concentration with PDW (Spearman r = 0.360, *p* = 0.013), D-dimer concentration (Spearman r = 0.513, *p* = 0.003) and fibrinogen (Pearson r = 0.456, *p* = 0.029) was recorded.

## 4. Discussion

In this study, we examined the level of parameters of oxidative stress and antioxidant protection, as well as the level of parameters of hemostasis and inflammation, in patients with COVID-19. Also, we evaluated the potential association of the redox status with the parameters of inflammation and hemostasis.

Oxidative stress represents an imbalance between oxidant production and antioxidant protection, which leads to cell damage, including lipid peroxidation and oxidation of DNA molecules [21]. It is found in many chronic diseases such as diabetes mellitus, coronary heart disease, and tumors, but also in some infections [22]. A large number of studies have shown that COVID-19 patients exhibit oxidative stress and an inhibition of the activity of the antioxidant system [23,24]. Respiratory infections are generally associated with cytokine production, inflammation, and redox imbalance [22]. Bastin et al. found a significantly increased level of oxidative stress parameters in patients with severe COVID-19 compared to a group that had a milder form of the disease [24]. In accordance with their results, our research showed that patients with COVID-19 have high levels of oxidative stress parameters compared to healthy subjects. Although all analyzed parameters of oxidative stress were higher in patients with COVID-19, a statistically significant increase in the concentration of H_2_O_2_ and TBARS was recorded. In addition, patients with COVID-19 showed a lower level of analyzed antioxidant parameters. The most significant difference in antioxidant parameters between the two groups of participants was found for CAT activity. In contrast, Lage et al. found higher CAT and SOD activity in the plasma of COVID-19 patients compared to healthy controls [25], while Yaghoubi et al. found no significant difference in the activities of these parameters in patients with COVID-19 [26].

Similarly, we estimated widespread inflammation with an increase in the total number of leukocytes, an increase in neutrophils and a decreased number of lymphocytes, as well as an increase in the concentration of C-reactive protein (CRP) and procalcitonin (PCT) in our patients. In a study conducted by Saberi-Movahed et al., hypoxia and higher CRP concentrations were associated with a poor prognosis of COVID-19 and a higher mortality rate. In addition, they reported that both platelet and lymphocyte count might be significant predictive markers of poor prognosis in COVID-19 patients [27]. NLR and PLR, as established markers of inflammation, were also higher in patients with COVID-19 compared to the control subjects in our study. It is especially important to understand that oxidative stress and inflammation are mutually reinforcing and together contribute to disease severity [28]. Bearing this in mind, we evaluated the relationship of pro-oxidant/antioxidant parameters with markers of inflammation in patients with COVID-19 and healthy subjects. It was shown that the parameters of oxidative stress (H_2_O_2_) were positively correlated with the level of inflammation (NLR). In addition to pro-oxidative parameters, antioxidant status parameters (SOD activity) showed a positive correlation with the degree of inflammation (NLR and PLR) in the group of patients with COVID-19. Our results were consistent with previous research showing that a high NLR was associated with very high production levels of oxidative stress parameters [29].

It is highly likely that COVID-19 is associated with a hypercoagulable state. However, platelet count showed no significant difference between the studied groups, while platelet indices, MPV and PDW, were statistically higher in those with COVID-19. Similarly, Aydınyılmaz et al. showed that severe patients with COVID-19 often exhibit thrombocytopenia with increased MPV and PDW [30]. A systematic review by Ligi and colleagues found that as many as 75% of studies reported significantly elevated VAT values in cohorts infected with COVID-19 compared to healthy controls [31]. Such evidence suggests that the reactivity of platelets in inflammatory and prothrombotic responses during SARS-CoV-2 infection is reflected through morphofunctional changes, i.e., an increase in platelet indices [31,32]. Some researchers even report the presence of the SARS-CoV-2 genome in the platelets of COVID-19 patients [13,33]. Large platelets are hemostatically more reactive and produce greater amounts of cytokines and prothrombotic factors, which can lead to thrombotic complications [34]. In addition, earlier studies reported increased D-dimer levels, increased fibrinogen concentrations, mildly prolonged prothrombin time (PT) and activated partial thromboplastin time (aPTT), which were consistent with our results [14,35,36].

In addition, hemostasis parameters also showed a relationship with pro-oxidative parameters. NO_2_^−^ showed a negative correlation with the number of platelets as well as a significant positive relationship with PDW. On the other hand, the parameters of the antioxidant system did not show significant correlations with the hemostasis parameters. Finally, our study showed a positive correlation between inflammation parameters (CRP and PCT) and hemostasis parameters (PT, aPTT, D-dimer and fibrinogen). Also, NLR and PLR positively correlated with PT length and D-dimer and fibrinogen concentration.

**Figure 11:**
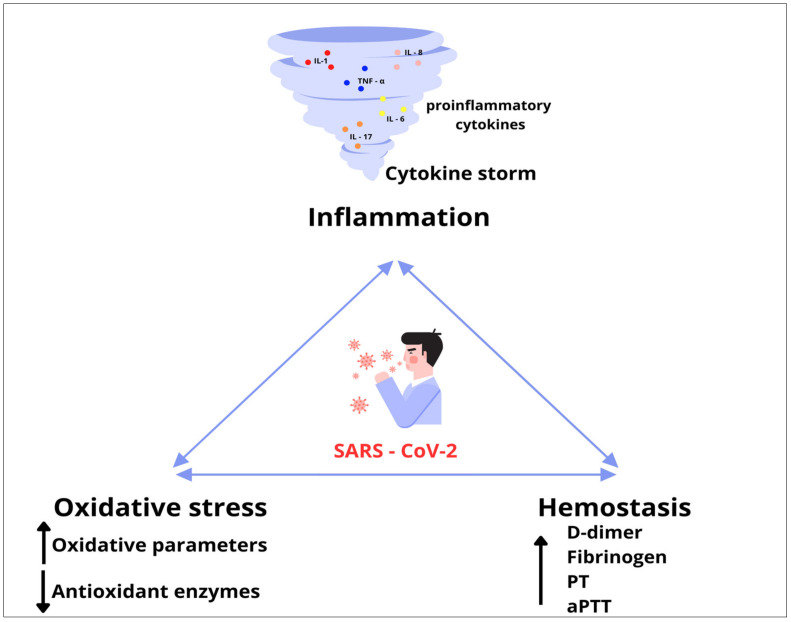
A vicious cycle of oxidative stress, inflammation and thrombosis.

It is already known that severe disease can cause a procoagulant state due to immobilization, mechanical ventilation and central venous access. However, SARS-CoV-2 can induce a hypercoagulable state by mechanisms unique to the virus, as well as the interrelationship between oxidative stress, inflammation and thrombosis [37]. A growing body of research confirms that the combination of overproduction of reactive ROS and hyperinflammation during SARS-CoV-2 can cause damage to the endothelial layer, ultimately causing endothelial dysfunction [38]. Endothelial dysfunction increases blood clotting and microthrombi formation. A SARS-CoV-2-induced prothrombotic state is mainly manifested by microthrombotic events known as immunothrombosis [39]. At the same time, inflammation and thrombosis cause a re-formation of ROS, which creates a vicious cycle of oxidative stress, inflammation and thrombosis with disease progression [40] (Figure 11).

Our study has a few limitations such as its cross-sectional design and the single-time blood sampling with no prospective patient follow-up. Some further research would be necessary to confirm the obtained results and give a more detailed picture of the pathophysiology of SARS-CoV-2.

In conclusion, hospitalized patients with COVID-19 have significantly higher levels of oxidative stress and significantly higher concentrations of parameters of inflammation and hemostasis compared to healthy subjects. There is a positive correlation between changes in hemostatic parameters and an increase in inflammatory markers in COVID-19 patients.

## Figures and Tables

**Table 1:** Baseline characteristics of patients with COVID-19 and healthy controls.

			COVID-19 Patients *n* = 105	Control Subjects *n* = 65
Gender	male		65 (61.9%)	42 (64.6%)
	females		40 (38.1%)	23 (35.4%)
Age (mean ± SD)			59.6 ± 14.78	58.91 ± 11.71
Duration of hospitalization			12.5 ± 7.9	-
Comorbidities		Diabetes mellitus type II	27 (25.7%)	-
		Hypertension	34 (32.4%)	-
		COPD	15 (14.3%)	-

Abbreviations: COPD, chronic obstructive pulmonary disease.

**Table 2:** Hematological and biochemical parameters in patients with COVID-19 and control subjects.

Parameter	COVID-19 Patients		Control Subjects		Significance *
	Mean ± SD	Min–Max	Mean ± SD	Min–Max	
Leukocyte (×10^9^/L)	9.66 ± 4.35	2.98–26.84	7.24 ± 1.53	4.80–9.90	*p* < 0.001
Neutrophils (×10^9^/L)	7.12 ± 4.12	1.44–25.02	4.60 ± 1.40	2.15–7.89	*p* < 0.001
Lymphocytes (×10^9^/L)	1.6 ± 0.97	0.21–4.64	1.97 ± 0.60	0.70–3.30	*p* = 0.007
Erythrocytes (×10^12^/L)	4.41 ± 0.71	2.37–6.18	4.47 ± 0.45	3.67–5.70	*p* = 0.080
Hemoglobin (g/L)	129.05 ± 24.39	73–179	141.88 ± 10.41	188–167	*p* < 0.001
Hematocrit (L/L)	0.39 ± 0.07	0.23–0.54	0.43 ± 0.035	0.35–0.50	*p* < 0.001
Platelets (×10^9^/L)	250.75 ± 83.52	54–482	240.16 ± 64.26	151–401	*p* = 0.463
MPV (fl)	7.41 ± 1.51	6.90–13.20	8.66 ± 0.71	7.40–10.30	*p* < 0.001
PDW (ratio)	16.31 ± 2.20	9.30–21.70	16.32 ± 0.60	14.50–17.10	*p* = 0.002

Abbreviations: MPV, mean platelet volume; PDW, platelet distribution width. * Statistically significant differences are bolded

## Data Availability

All data and material supporting this study will not be publicly available due to ethical restrictions, but will be available upon reasonable request and with the author’s consent.
